# Rapid Detection of *Listeria* by Bacteriophage Amplification and SERS-Lateral Flow Immunochromatography

**DOI:** 10.3390/v7122962

**Published:** 2015-12-14

**Authors:** Nicholas R. Stambach, Stephanie A. Carr, Christopher R. Cox, Kent J. Voorhees

**Affiliations:** Department of Chemistry and Geochemistry, Colorado School of Mines, Golden, CO 80401, USA; nstambac@mines.edu (N.R.S.); scarr@mines.edu (S.A.C.); crcox@mines.edu (C.R.C.)

**Keywords:** lateral flow immunochromatography, *Listeria monocytogenes*, surface-enhanced Raman spectroscopy, A511, phage amplification

## Abstract

A rapid *Listeria* detection method was developed utilizing A511 bacteriophage amplification combined with surface-enhanced Raman spectroscopy (SERS) and lateral flow immunochromatography (LFI). Anti-A511 antibodies were covalently linked to SERS nanoparticles and printed onto nitrocellulose membranes. Antibody-conjugated SERS nanoparticles were used as quantifiable reporters. In the presence of A511, phage-SERS nanoparticle complexes were arrested and concentrated as a visible test line, which was interrogated quantitatively by Raman spectroscopy. An increase in SERS intensity correlated to an increase in captured phage-reporter complexes. SERS limit of detection was 6 × 10^6^ pfu·mL^−1^, offering detection below that obtainable by the naked eye (LOD 6 × 10^7^ pfu·mL^−1^). Phage amplification experiments were carried out at a multiplicity of infection (MOI) of 0.1 with 4 different starting phage concentrations monitored over time using SERS-LFI and validated by spot titer assay. Detection of *L. monocytogenes* concentrations of 1 × 10^7^ colony forming units (cfu)·mL^−1^, 5 × 10^6^ cfu·mL^−1^, 5 × 10^5^ cfu·mL^−1^ and 5 × 10^4^ cfu·mL^−1^ was achieved in 2, 2, 6, and 8 h, respectively. Similar experiments were conducted at a constant starting phage concentration (5 × 10^5^ pfu·mL^−1^) with MOIs of 1, 2.5, and 5 and were detected in 2, 4, and 5 h, respectively.

## 1. Introduction

*Listeria monocytogenes* is a Gram-positive, motile, facultative anaerobic rod and the etiological agent of food-borne listeriosis. Symptoms of listeriosis include gastroenteritis, diarrhea, meningitis and bacteremia. It also contributes significantly to spontaneous miscarriages [1]. Listeriosis is responsible for approximately 1600 food-related illnesses and 260 deaths in the U.S. annually [2], and is the third leading cause of death among foodborne pathogens (behind *Salmonella* spp. and *Toxoplasma gondii*), with 94% of cases leading to hospitalization and a mortality rate of 20%–30% [2,3]. The U.S. has adopted a zero tolerance policy for *Listeria* on food. However, because of the protracted turnaround times (TATs) of conventional detection methods, the majority of food products potentially contaminated with *L. monocytogenes* are not tested before entering the marketplace, increasing the risk of widespread outbreaks. Given *Listeria’s* natural occurrence in soil and among animal reservoirs, many processed foods are at risk of *Listeria* contamination. These include ready-to-eat meats and cheeses, unpasteurized dairy products, hot dogs, smoked seafood and raw produce [4]. Further adding to difficulties in outbreak prevention is *Listeria’s* ability to grow over a wide range of temperatures, including those as low as 1 °C, which allows it to propagate even when refrigerated (~4 °C). One example of this is the 2011 Jensen Farms outbreak associated with contaminated cantaloupes [5]. One hundred forty-seven people were infected across 28 states, resulting in 33 deaths and one miscarriage, making it the second deadliest food-borne related outbreak in U.S. history.

The most commonly employed *Listeria* detection/differentiation methods include culture-based biochemical tests (such as the Remel MICRO-ID^®^ *Listeria*, bioMérieux *Listeria* API^®^ test system and VITEK^®^ 2 Compact), and the Christie, Atkins, Munch-Petersen (CAMP) test. These commercially available tests have average TATs of 4–6 days [6,7]. Polymerase chain reaction (PCR)-based methods, such as the DuPont BAX^®^ system, offer an alternative for rapid detection; however, DNA amplification can be negatively impacted by polymerase inhibitors found in food components, leading to false negatives [8]. Another limitation with PCR is the inability to differentiate between live and dead cells, a critical issue in the food industry due to the fact that many food products undergo treatment to kill bacteria [9].

The Jensen Farms outbreak and others that have occurred since 2011 strongly suggest the need for more rapid detection methods for food-borne *Listeria*. This study exploited *Listeria* phage A511 and its natural, rapid and host-specific amplification. A511, a large (134,494 kbp), well-characterized myovirus was chosen for this study because of its lytic nature, broad-host range, and ability to produce many progeny phages (burst size 40–50) in a short amount of time, with a latent period of 55–60 min at 30 °C [10,11,12]. While only genus-specific, A511 still proves useful in detection systems, as government regulatory agencies, such as the Food and Drug Administration, the US Department of Agriculture, and Food Standards Australia and New Zealand, do not differentiate between *Listeria* spp., because the presence of non-pathogenic *Listeria* spp. indicate poor hygiene and indicate conditions that promote the growth of pathogenic *L. monocytogenes* [13,14]. As such, A511 has been used in a variety of detection schemes [11,15,16,17]. Phages are predatory bacterial viruses that have long been utilized for bacterial detection and identification, and are simple and inexpensive to produce [9,18,19,20]. Fundamentally, phage amplification involves phage attachment to the surface of a host bacterium, followed by insertion of its nucleic acid. This results in hijacking of the host replication machinery, and the subsequent transcription and translation of phage genes. Replication and assembly of progeny phages follows, culminating in host cell lysis and release of new infectious virions (progeny phage) into the surrounding milieu to repeat the cycle. This leads to a rapid increase (termed burst) in phage concentration in less than 3 h. In phage amplification assays, such as the one described here, input phages are added below detection limits of the detection of choice and allowed to specifically infect target bacterial pathogen and multiply. An increase in the number of detectable progeny phages signals a successful amplification event, which therefore indicates the presence of the target bacteria. By employing contemporary analytical techniques, such as matrix-assisted laser desorption/ionization time of flight mass spectrometry [21,22,23], progeny phages have been used as secondary biomarkers for species-specific bacterial detection.

Alternatively, LFI offers a less expensive, rapid, point-of-need biosensor for phage-based bacterial detection. LFI combined with phage amplification (diagrammed in Figure S1) has previously been used for the rapid detection of *Staphylococcus aureus* and *Bacillus anthracis* [24,25,26]. The LFI system used in these assays employed colormetric particles for visual determination. The drawback to visual-based LFI systems lies in the difficulty in the reading of the test line at low analyte concentrations, where faint lines can be misread and lead to false negative results. Various reporter particles have been developed to address this issue and can include fluorescent, spectroscopic or contain enzymatically labeled particles [27,28,29].

LFI biosensors have been combined with spectroscopic techniques, such as SERS, in the past for detection of cancer antigens and influenza [28,30]. Figure S2 (Supplementary materials) shows a schematic of an SERS nanoparticle used in this study. Nanoparticles consisted of a gold core covered with a layer of an organic, Raman-active reporter dye, encased in a thin layer of silica and surface functionalized with thiol groups to facilitate antibody attachment [31,32]. The dye in the SERS nanoparticles produces a unique Raman spectrum to allow for identification and signal quantification. The use of several particle types, each producing unique Raman spectra, allows for the simultaneous detection of multiple target analytes [33]. While SERS particles at high concentrations allow visual observation of LFI test lines, test line visibility can be faint and unreliable at low concentrations. It is hypothesized that the use of SERS extends sensitivity below visual levels and provides a quantifiable signal, thus eliminating the need for visual conformation. Figure S3 (Supplementary materials) displays a characteristic spectrum of the organic reporter molecule *trans*-1,2-bis(4-pyridyl)-ethylene, which was used in this study [33].

In this work, we describe the development of a novel SERS-LFI device utilizing anti-A511 conjugated SERS nanoparticles. This was combined with phage amplification for rapid and specific detection of *L. monocytogenes*. A511 amplification experiments were monitored via SERS-LFI and confirmed by spot titer assay. Raman signal quantitation and overall test sensitivity is discussed. While the aim of this study was specifically for *Listeria* detection, this platform can be adapted to other bacterial pathogens for which a suitable lytic phage is available.

## 2. Materials and Methods

### 2.1. A511 Propagation

*Listeria ivanovii* ATCC 19119 was obtained from the American Type Culture Collection (Manassas, VA, USA) and used for A511propagation (provided by Martin Lossener, Institute of Food, Nutrition and Health, ETH Zurich, Zurich, Switzerland) using soft agar overlays [34]. Briefly, 80 μL aliquots of A511 (10^8^ pfu·mL^−1^) were spotted onto lawns of *L. ivanovii* mixed with soft agar (0.5% agar in Brain Heart Infusion, BHI) and were incubated at 23 °C for 24 h. Resulting plaques were harvested by an addition of 3 mL of phosphate buffered saline (PBS), pH 7.4, followed by centrifugation of the resulting slurries at 9000× *g* for 15 min at 4 °C. Supernatants were filter-sterilized with 0.22 μm PES 1000 mL Rapid Flow Filter Units (Nalgene, Rochester, NY, USA). Polyethylene glycol phage precipitation (PEG 8000) (OmniPur, Gibbstown, NJ, USA) was conducted as previously described [35]. Further phage purification was conducted by cesium chloride gradient ultrafiltration [35]. Residual CsCl was removed by dialysis in PBS. All anti-phage antibodies were prepared using this purified phage.

### 2.2. Production and Purification of Anti-A511 Antibodies

Polyclonal rabbit anti-A511 phage IgG antibodies were prepared by Antibodies Incorporated (Davis, CA, USA). Antibodies were Protein G purified (Nab^TM^, Thermo Scientific, Rockford, IL, USA) and specificity was confirmed by an enzyme-linked immunosorbent assay (ELISA). Purified antibodies were dialyzed in PBS, concentrated by ultrafiltration (Amicon^®^ Ultra, 30 kDa cutoff) (Millipore, Billerica, MA, USA) and filter-sterilized with 0.22 μm PES filters (Thermo Scientific).

### 2.3. Nanoparticle Reporter and Control Particle Preparation

Anti-phage SERS reporter particles were prepared by conjugation of 50–60 nm diameter SERS-S440 Nanotags (SERS NPs, OD 24) (Becton Dickinson, Research Triangle Park, NC, USA) with purified polyclonal anti-A511 antibodies. The conjugation method described here is a modification found in Wang *et al.* [36]. The crosslinker, sulfosuccinimidyl 4-[*N*-maleimidomethyl] cyclohexane-1-carboxylate (sulfo-SMCC) (Thermo Scientfic) was prepared in degassed conjugation buffer, 10 mM 3-morpholinopropane-1-sulfonic acid (MOPS) (Sigma Aldrich, St. Louis, MO, USA) (pH 7.2). Sulfo-SMCC was reacted at 50 molar equivalents of purified anti-A511 antibodies for 30 min at 23 °C. Stock SERS NPs were prepared by dilution into a degassed conjugation buffer at a volume ratio of 1:1, and then reacted with antibody-crosslinker complex solution (350 molar excess antibodies to SERS NPs) at 23 °C for 3 h with continuous inversion. After primary conjugation, unreacted thiols on SERS NPs were blocked by a solution of *N*-ethylmaleimide (NEM) (Thermo Scientific), and prepared in a degassed conjugation buffer (650,000 molar excess NEM to SERS NPs). Concomitantly, Blocker™ Casein in PBS (Thermo Scientific) was added to block the surface of the SERS NPs. Blocking was performed at 23 °C for 2 h with continuous inversion. Unreacted sulfo-SMCC malimide groups were quenched with 2-mercaptoethanesulfonic acid (MP Biomedicals, Santa Ana, CA, USA) at 23 °C with continuous inversion for 45 min. Excess reagents were removed by centrifugation (1000× *g* for 10 min), and the supernatant was removed and replaced with a storage buffer, 50 mM sodium borate (Fisher Scientific, Fairlawn, NJ, USA), 1% v·v^−1^ gelatin (telostean gelatin from cold water fish skin, Sigma Aldrich), and 0.05% w·v^−1^ sodium azide (Fisher Scientific) (pH 7.5). This was repeated 4 times and conjugated particles were stored at 4 °C. Figure S4 (see Supplementary materials) shows a schematic overview of the conjugation.

Control particles were prepared by passive conjugation of blue carboxy-modified polystyrene Seradyn particles (Thermo Scientific, Waltham, MA, USA, 223 nm diameter, 2.5% solids) to ImmunoPure^®^ biotinylated bovine serum albumin (Biotin-LC-BSA) (biotin BSA) (Thermo Scientific). Briefly, particles were centrifuged at 9000× *g* for 5 min, and the supernatant discarded. Particles were resuspended in 50 mM 2-[4-(2-hydroxyethyl)piperazin-1-yl]ethanesulfonic acid (HEPES) (Research Organics, Cleveland, OH, USA) (pH 7.4) with a 2 mg·mL^−1^ biotin BSA added at a 2.5:1 ratio and mixed with continuous inversion for 90 min at 23 °C. The resulting suspension was centrifuged at 10,000× *g* for 5 min, the supernatant discarded, and the pellet resuspended in HEPES. This was repeated twice with final resuspension in TNGA (0.025 M Tris base, 0.1 M NaCl, 1% v·v^−1^ fish gelatin, and 0.05% w·v^−1^ sodium azide) (pH 8.4) and blocked for 1 h at 23 °C with continuous inversion. Particles were pelleted by centrifugation (10,000× *g*, 5 min) with the supernatant removed and replaced with fresh TNGA. This centrifugation step was repeated twice more, then resuspended to 0.625% solids and stored at 4 °C.

### 2.4. LFI Device Fabrication

Nitrocellulose membranes (Millipore Hi-Flow 180, Billerica, MA, USA, SHF1800425) were prepared by applying purified anti-A511 antibodies as a test line and NeutrAvidin™ Biotin-Binding Protein (Thermo Scientific) as a control line using an IVEK Digispense 2000 striper (IVEK Corporation, North Springfield, VT, USA). Anti-A511 (2 mg·mL^−1^) and NeutrAvidin (1.25 mg·mL^−1^) were applied at a rate of 4 μL·s^−1^ and dried for 15 min at 35 °C. Antibody/control line-stripped membranes were stored desiccated at 23 °C. Release pads (Schleicher & Schuell, Keene, NH, USA) were prepared by impregnation of glass fiber release medium with a solution of SERS reporter nanoparticles (0.02% solids) and control particles (0.01% solids) in 2 mM sodium borate, 0.1 M NaCl, 1% v·v^−1^ fish gelatin, 0.05% w·v^−1^ sodium azide, and 3% w·v^−1^ sucrose (Baker, Phillipsburg, NJ, USA) (pH 8.4) dried for 30 min at 35 °C and stored desiccated at 23 °C until assembly.

LFI devices were fabricated by mating sample pad, release pad, nitrocellulose membrane, and absorbing pad (Schleicher & Schuell) to an adhesive backboard (G&L Precision Die Casting, San Jose, CA, USA) with an overlap of approximately 2 mm between layers (Figure S1). Assembled LFI strips were cut to a width of 3.7 mm using a programmable sheer (Kinematic Automation Inc., Twain Harte, CA, USA) and desiccated at 23 °C until use.

### 2.5. Determination of LFI Limit of Detection

LFI LOD was determined by a serial dilution of filter-sterilized A511 in tryptose and 1 mM CaCl_2_. Triplicate analysis of phage dilutions ranging from 1 × 10^9^ plaque forming units (pfu)·mL^−1^ to 2 pfu·mL^−1^ were tested, in addition to phage-free controls. Prior to application, phage samples were mixed with a running buffer at a 1:1 ratio to a volume of 100 μL and applied drop-wise to the sample pad. The running buffer consisted of 0.1 M sodium borate, 3% w·v^−1^ bovine serum albumin (BSA) (Sigma Aldrich), and 1% v·v^−1^ Tween^®^ 20 (Sigma Aldrich) (pH 8). LFI was conducted for 30 min, wicking pads were removed to prevent backflow, and LFI strips were dried in a desiccator for 10 min. Test lines were interrogated by Raman spectroscopy (Advantage 785, DeltaNu Inc., Laramie, WY, USA) at 785 nm with a laser power of 51 mW. Twelve measurements were collected along the test line with an interrogation time of 3 s per measurement. Whole spectra analyses of SERS samples were performed by assuming that sample spectra were a linear combination of an SERS nanoparticle reference spectrum and a nitrocellulose reference spectrum, which was then solved using least squares [28,37].

### 2.6. Phage Amplification and LFI Analysis

Four phage amplification experiments were performed using A511 and *L. monocytogenes* ATCC 19115 at a multiplicity of infection (MOI) of 0.1 with varying A511 starting concentrations (1 × 10^6^, 5 × 10^5^, 5 × 10^4^, and 5 × 10^3^ pfu·mL^−1^). Two additional phage amplification experiments were conducted at MOIs of 2.5 and 5 with the same starting phage concentration (5 × 10^5^ pfu·mL^−1^). The A511 starting concentration was always below the determined phage LOD to avoid false positive results caused by initial infecting phage. Overnight cultures of *L. monocytogenes* were back-diluted and grown to an OD_620_ of 0.3, corresponding to 1 × 10^8^ colony forming units (cfu) mL^−1^, and were subsequently diluted to the appropriate starting concentration at a 10 mL final volume. A511 (5 × 10^7^ pfu·mL^−1^) was added at appropriate volumes for the desired starting phage concentrations. Aliquots were taken from amplification reactions every hour and filtered to remove bacteria. Fifty microliters of resulting filtrates were mixed with 50 μL running buffer and applied to LFI strips as described in the previous section. Time was required to run the LFI device (~30 min), dry the strip (~10 min) and interrogate the LFI strip with the Raman spectrometer (~5 min). Phage concentration was also followed in parallel for the duration of the experiment by spot titer assay as previously described [38] to confirm results of the SERS-LFI.

## 3. Results and Discussion

### 3.1. SERS-LFI Device Optimization

Phage biology, SERS, and LFI are independent concepts previously utilized for bacterial detection [39,40,41,42,43]. The current study combined aspects of each of these principles to overcome obstacles associated with manufacturing reliable SERS-LFI devices. Consistent, optimized antibody conjugation to SERS NPs and LFI construction were particularly important to reproducibility; thus, improving these methods was a major focus of the current study. All changes to the original NP manufacturer’s conjugation protocols were intended to minimize agglomeration of conjugated NPs on the membrane. Agglomerated NPs did not consistently travel down the nitrocellulose, which created an artificially high signal with phage-free controls. Problems addressed in this paper included: the optimization of conjugation and running buffer composition; the antibody:SERS NP ratio; the blocking of non-specific phage-particle interactions; the capture antibody application; the membrane flow rate; and the SERS reporter release pad concentration. Troubleshooting these parameters led to the development of a new, robust protocol for fabrication of SERS-LFI devices. These adjustments are summarized in Table 1, and further discussion can be found in the Supplementary materials.

**Table 1:** Summary of conjugation and LFI parameters investigated.

Parameter Investigated	Optimized Conditions
Conjugation Buffer	10 mM MOPS, pH 7.15
Running Buffer	0.1 M sodium borate, 3%, 1% Tween 20, pH 8
Antibody:SERS NP ratio	350:1
Thiol blocker	*N*-ethylmaleimide
Capture antibody concentration	2 mg/mL
Membrane Flow Rate	180 mm/4 s
SERS reporter release pad concentration	0.02% solids

### 3.2. Determination of LFI Limit of Detection

**Figure 1:**
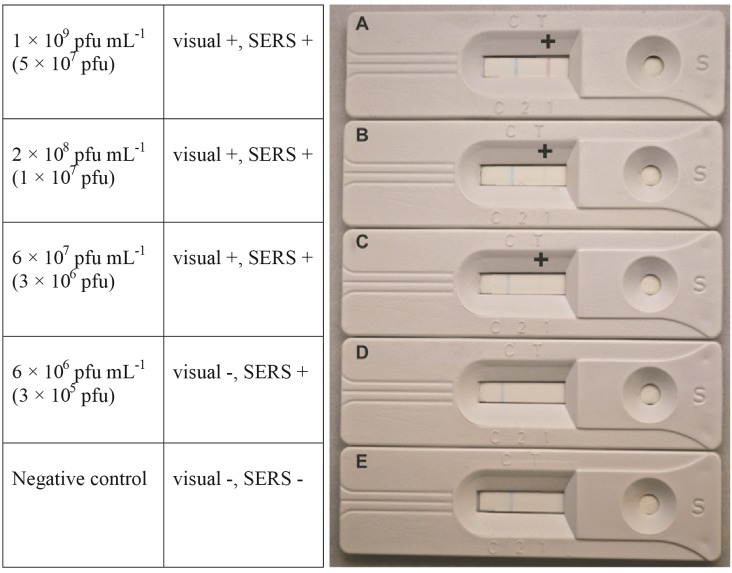
Visual LOD was determined by the appearance of a pink line at the test line. The column on the left represents the phage concentration sampled, while phage number is indicated in the parenthesis.

Both visual and spectroscopic limits of detection were determined for prototype SERS-LFI. Visual positives were determined by the formation of a pink test line. As shown in Figure 1A,B, phage concentrations of 1 × 10^9^ pfu·mL^−1^ and 2 × 10^8^ pfu·mL^−1^ produced clearly visible pink lines. The minimum concentration for which a visually observable colored line formed was 6 × 10^7^ pfu·mL^−1^ (Figure 1C). It should be noted that this concentration produced a very weak response, which could be mistaken as a negative result. The possibility of such outcomes emphasizes the difficulty associated with qualitative visual analysis and interpretation by the untrained user.

**Figure 2:**
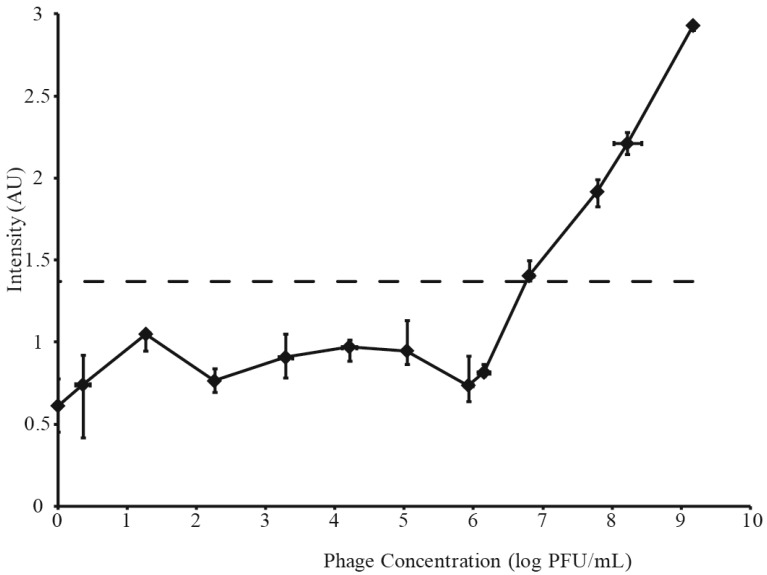
Phage concentration *vs.* Raman intensity for dilution of phage. The SERS LOD is indicated by a dashed line.

**Figure 3:**
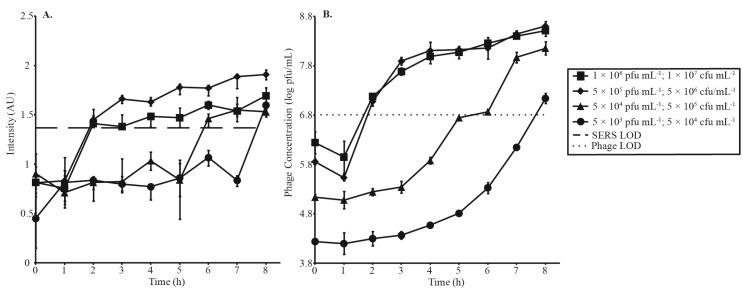
Four phage amplification experiments carried out at MOI 0.1 and decreasing concentrations of phage and bacteria to investigate the time needed for a positive detection, monitored by (**A**) SERS-LFI and (**B**) parallel spot titer assay. SERS and phage LODs are represented by a dashed and dotted line, respectively.

**Figure 4:**
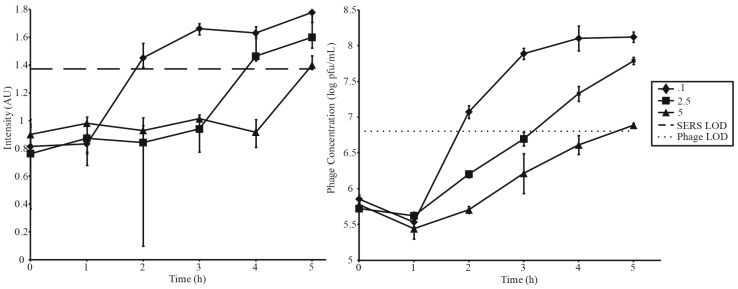
Phage amplifications carried out at 3 different MOIs utilizing a constant starting phage concentration of 5 × 10^5^ pfu·mL^−1^. SERS and phage LODs are represented by a dashed and dotted lines respectively.

Raman signals measured from the same serial dilutions of A511 are shown in Figure 2. These intensities represent the interquartile range (IQR) of 12 random shots along a single test line, shown by the vertical error bars. The IQR was necessary, because heterogeneous membrane pore distribution was observed to lead to areas of higher and lower Raman intensity along the length of the test line. Horizontal error bars in Figure 2 represent standard deviation of phage titers done in triplicate. The lowest phage dilution to produce a Raman signal above phage-fee controls was 6× 10^6^ pfu·mL^−1^. Thus, this phage dilution, represented by a dotted line in Figure 3B and Figure 4B, and the resulting Raman signal (intensity of 1.37), represented by a dashed line in Figure 3A and Figure 4B, were accepted as the phage and SERS LOD, respectively. A comparison between SERS and visual limits of detection (6 × 10^6^ pfu·mL^−1^ and 6 × 10^7^ pfu·mL^−1^, respectively) demonstrated an order of magnitude greater sensitivity for SERS. All IQR measurements below SERS LOD represent instrument noise.

### 3.3. Phage Amplification and SERS-LFI Analysis

Four phage amplification experiments at a MOI of 0.1 were monitored by SERS-LFI (Figure 3A, Table 1) and parallel spot titer assay (Figure 3B). The purpose of this experiment was to investigate how decreasing phage and bacterial concentrations affected the time it took to get a positive result. Phage concentrations were varied from 1 × 10^6^ pfu·mL^−1^ to 5 × 10^3^ pfu·mL^−1^, and corresponding bacterial concentrations were varied from 1 × 10^7^ cfu·mL^−1^ to 5 × 10^4^ cfu·mL^−1^. SERS detection of the highest phage concentrations (1 × 10^6^ pfu·mL^−1^ and 5 × 10^5^ pfu·mL^−1^) was achieved in 2 h. Additional time was required before decreased phage concentrations reached detectable levels (6 h for 5 × 10^4^ pfu·mL^−1^ and 8 h for 5 × 10^3^ pfu·mL^−1^). Parallel spot titer assays confirmed that all positive tests represented phage concentrations greater than the established LOD and that increased SERS intensities correlated with phage amplification.

A second series of amplifications was conducted to investigate the relationship between MOI and detection time (Figure 4, Table 2). As previously discussed, an MOI of 0.1 resulted in detection in 2 h. Detection times increased with increasing MOIs. MOIs of 2.5 and 5 surpassed detection limits at 4 h and 5 h, respectively. Parallel spot titer assays are shown in Figure 4B. Error bars in Figure 3A and Figure 4A correspond to the IQR of 12 random shots along the test line of a single LFI strip, while error bars in Figure 3B and Figure 4B represent standard deviation of phage titers measured in triplicate. Table 2 summarizes Figure 3 and Figure 4.

**Table 2:** Summary of SERS-LFI coupled with phage amplification detection times.

**Phage Amplification Series 1**			
**Phage Concentration (pfu·mL^−1^)**	**Bacterial Concentration (cfu·mL^−1^)**	**MOI**	**SERS-LFI Detection Time (h)**
1 × 10^6^	1 × 10^7^	0.1	2
5 × 10^5^	5 × 10^6^	0.1	2
5 × 10^4^	5 × 10^5^	0.1	6
5 × 10^3^	5 × 10^4^	0.1	8
**Phage Amplification Series 2**			
**Phage Concentration (pfu·mL^−1^)**	**Bacterial Concentration (cfu·mL^−1^)**	**MOI**	**SERS-LFI Detection Time (h)**
5 × 10^5^	1 × 10^6^	0.1	2
5 × 10^5^	2 × 10^5^	2.5	4
5 × 10^5^	1 × 10^5^	5	5

The results displayed in Figure 3 and Figure 4 highlight a drawback of phage amplification assays. Low concentrations of phage and bacteria decrease the chance of a phage and a bacterium meeting, irrespective of the binding efficiency of the phage to the bacterium [39]. This increases the time necessary for the phage concentrations to reach the detection limit of the detector device [44,45,46]. Hagens and Loessner have previously discussed this phenomenon [47].

In conclusion, this is the first report of phage amplification combined with the use of SERS and LFI for *Listeria* detection. This study focused on establishing a robust anti-phage conjugation protocol and on optimization of LFI construction, with the goal of minimizing nanoparticle agglomeration and improving reproducibility. The resulting devices are capable of detecting progeny A511 at concentrations as low as 6× 10^6^ pfu·mL^−1^. The shortest detection time for *L. monocytogenes* was 2 h, while, in a separate experiment, detection of bacteria at a concentration of 1 × 10^4^ cfu·mL^−1^ was achieved in 8 h. While an enrichment step is still needed to obtain bacterial concentrations that allow for propagation of progeny phage to detectable levels, phage amplification eliminates the need for downstream plating on selective media, and for further biochemical or molecular tests (reducing detection by 24–48 h), while providing evidence of viable cells. SERS-LFI allows for positive identification in as little as 30 min, while traditional plaque assays can take up to 24 h.

## Supplementary Files

Supplementary File 1
