# Supplementary material for: Rapid Detection of Listeria by Bacteriophage Amplification and SERS-Lateral Flow Immunochromatography
Source: Viruses. 2015 Dec 14;7(12):6631–41. doi: 10.3390/v7122962 (PMC4690885; doi:10.3390/v7122962)
Supplement: Supplementary File 1 [file viruses-07-02962-s001.pdf]

Supplementary Materials: Rapid Detection of *Listeria* by Bacteriophage Amplification and SERS-Lateral Flow Immunochromatography

Nicholas R. Stambach, Stephanie A. Carr, Christopher R. Cox and Kent J. Voorhees *

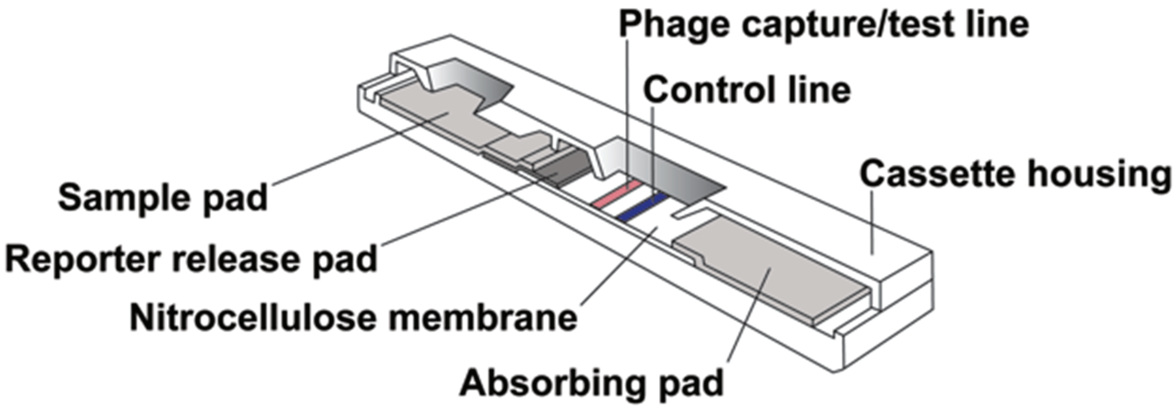

Figure S1. Schematic of phage-based LFI device.

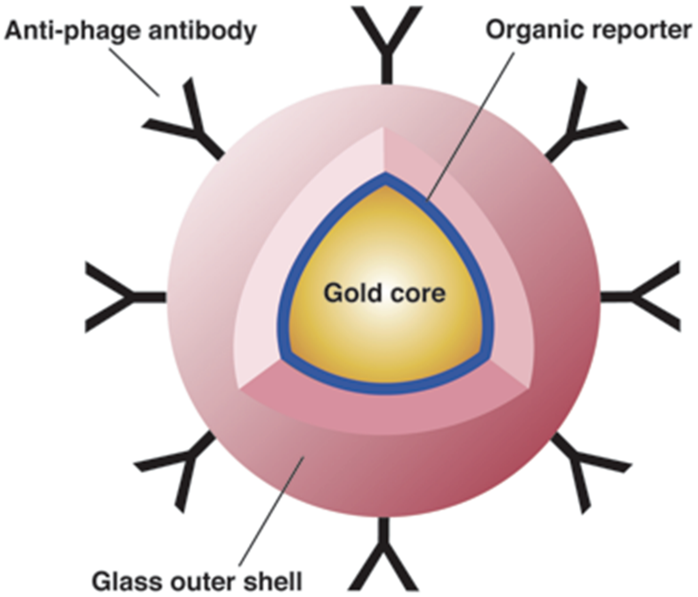

Figure S2. Schematic of an antibody-linked SERS NP.

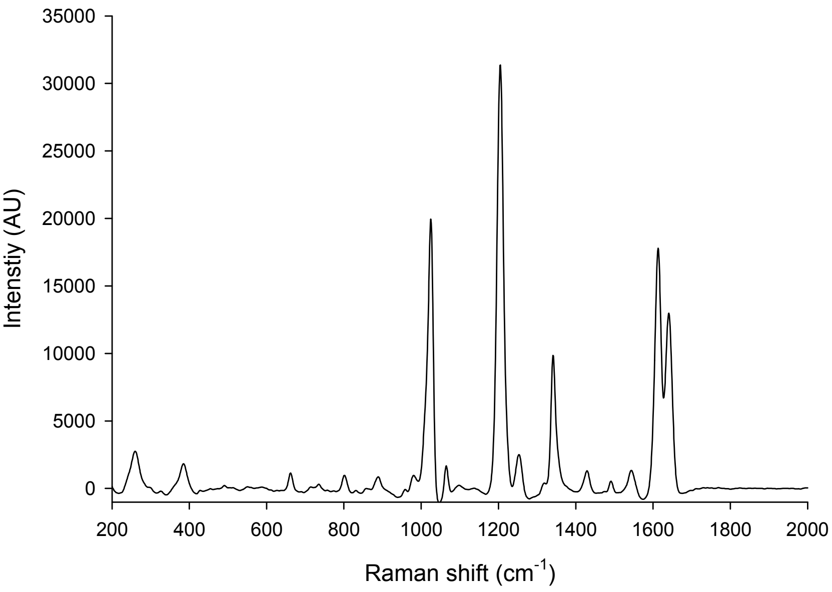

Figure S3. Raman spectrum of *trans*-1,2-bis(4-pyridyl)-ethylene, SERS NP S440.

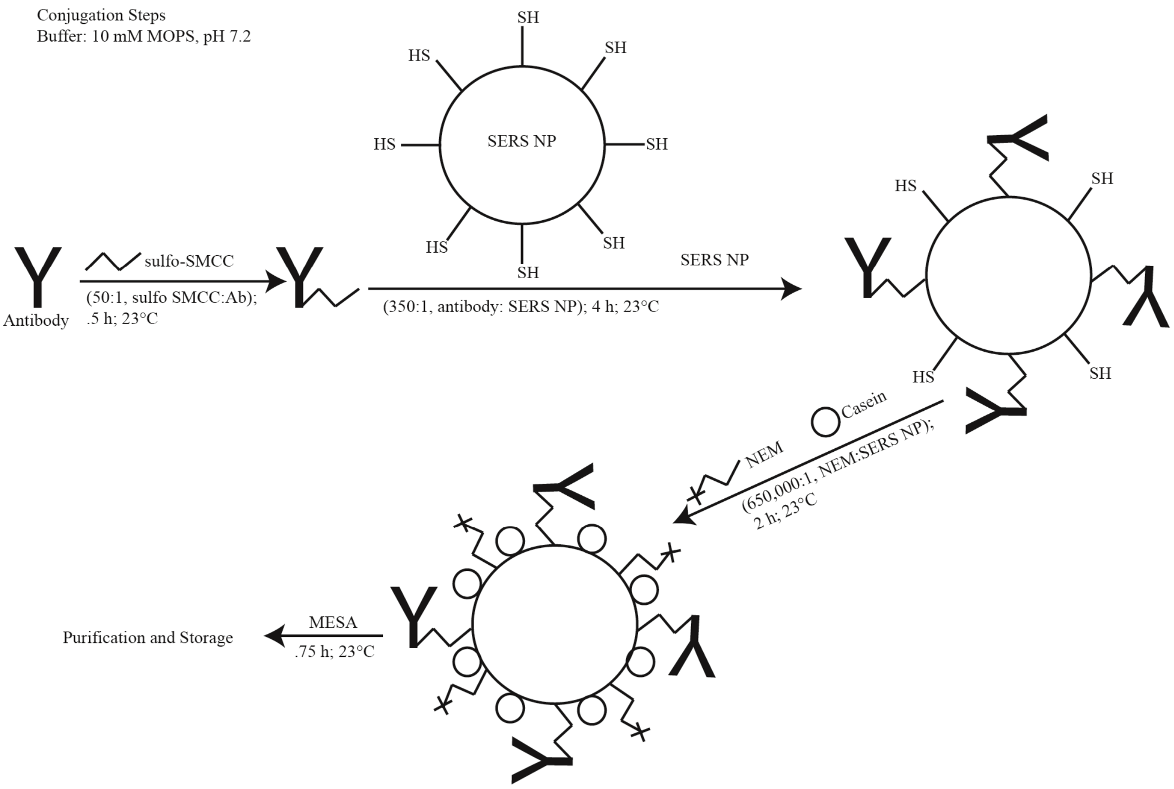

Figure S4. Schematic illustration of anti-phage antibody to SERS NP.

Discussion of Optimized Parameters

Original manufacturer instructions called for a 50 mM phosphate (pH 7.15) solution; however, we observed that this caused an aggregation of NPs during conjugation. A 10 mM MOPS (pH 7.15) conjugation buffer was observed to minimize agglomeration. Precise antibody:SERS NP molar ratios were also critical for preventing agglomeration. High antibody:NP molar ratios (above 350:1) increased phage-free control signal, and even higher ratios (above 700:1) gave rise to faint visual false positives. Molar ratios below 350:1 gave less sensitivity but minimized false positives with phage-free controls. The blocking of free reactive thiols on NPs with NEM further prevented agglomeration and particle precipitation during conjugation.

Once particle conjugation was optimized, maximal LFI running conditions were investigated. First, the running buffer composition was examined. The running buffer solubilized both reporter and control particles in the release pad and served as a mobile phase to carry particles down the membrane. A 50 mM HEPES, 0.05% Tween (pH 8) solution was initially used; however, it did not adequately transport the NPs through the LFI device to the absorbing pad, resulting in heterogeneous distribution of particles throughout the membrane. The running buffer used in [1], which consisted of 0.1 M sodium borate, 3%, 1% Tween 20, pH 8, was examined with phage-free controls and observed to give the least non-specific binding. Particles were also observed to completely transit the membranes to the wicking pads.

Other factors that were investigated included LFI capture antibody concentration, membrane flow rate and NP release pad concentration. Capture antibody application concentration was optimal at 2 $mg \cdot mL^{-1}$. Concentrations higher than 2 $mg \cdot mL^{-1}$ resulted in lower overall device sensitivity. A manufacturer-determined, membrane-limited capillary flow rate of 180 $s \cdot 4\ mm^{-1}$ was observed as optimal, while rates of 120 or 90 $s \cdot 4\ mm^{-1}$ decreased sensitivity. SERS NP release pad concentration also affected Raman sensitivity. A final concentration of 0.02% solids SERS NPs was determined to be optimal. Higher particle concentrations caused an increased in phage-free control false positive signal, while lower concentrations decreased sensitivity.

Reference

1. Noble, J.; Attree, S.; Horgan, A.; Knight, A.; Kumarswami, N.; Porter, R.; Worsley, G. Optical scattering artifacts observed in the development of multiplexed surface enhanced Raman spectroscopy nanotag immunoassays. *Anal. Chem.* **2012**, *84*, 8246–8252.

© 2015 by the authors; licensee MDPI, Basel, Switzerland. This article is an open access article distributed under the terms and conditions of the Creative Commons by Attribution (CC-BY) license (http://creativecommons.org/licenses/by/4.0/).
